# Transcriptomic and Epigenetic Alterations in Dendritic Cells Correspond With Chronic Kidney Disease in Lupus Nephritis

**DOI:** 10.3389/fimmu.2019.02026

**Published:** 2019-08-27

**Authors:** Anna Wardowska, Michał Komorniczak, Barbara Bułło-Piontecka, M. Alicja Dȩbska-Ślizień, Michał Pikuła

**Affiliations:** ^1^Laboratory of Tissue Engineering and Regenerative Medicine, Department of Embryology, Medical University of Gdansk, Gdansk, Poland; ^2^Department of Clinical Immunology and Transplantology, Medical University of Gdansk, Gdansk, Poland; ^3^Department of Nephrology, Transplantology, and Internal Diseases, Medical University of Gdansk, Gdansk, Poland

**Keywords:** dendritic cells, DNA methylation, histone modifications, interferon regulatory factors, chronic kidney disease, systemic lupus erythematosus

## Abstract

Systemic lupus erythematosus (SLE) is a serious autoimmune disease with variety of organ manifestations. The most dreadful one, affecting the majority of SLE patients, is kidney manifestation—lupus nephritis (LN). Dendritic cells (DC) are believed to be one of the culprits of immune dysregulation in LN. Flow cytometry analysis was applied to identify the frequency and activity of peripheral blood DCs subpopulations: myeloid and plasmacytoid, in LN patients. Magnetically isolated mDCs and pDCs were subjected to molecular analysis of genes expression, evaluation of global DNA methylation and histone H3 methylation. We observed distinctive features of DCs associated with the stages of nephritis in LN patients. Lower numbers of pDCs were observed in patients with severe LN, while increased co-stimulatory potential of mDCs was connected with the early, mild stage of this disease. IRF1 transcript upregulation was specific for mDCs from total LN patients, while exceptional amount of IRF1 mRNA was detected in mDCs from severe LN patients. DCs DNA hypermethylation seemed characteristic for severe LN, whereas a decrease in H3K4me3 and H3K27me3 marks was significant for the early stages of LN. These findings present dendritic cell alterations that may reflect renal involvement in SLE, laying foundations for new strategy of diagnosis and monitoring of LN patients, omitting invasive kidney biopsies.

## Introduction

Autoimmunity refers to abnormalities in the activity and function of the immune system leading to the deleterious effect of a loss of tolerance to self-antigens. The autoimmune response can manifest through widespread and multiorgan dysfunction resulting in systemic diseases such as systemic lupus erythematosus (SLE). SLE is a chronic disease characterized by intermittent episodes of symptoms augmentation imposing immunosuppressive treatment (1). The hallmark of SLE is the presence of autoantibodies directed against nuclear antigens, that form immune complexes (IC), preferably deposited in affected tissues and organs (2). Among the various organ manifestations, the renal involvement, referred as lupus nephritis (LN), is the most feared, as it significantly increases the morbidity and mortality in SLE patients. LN, an IC-mediated glomerulonephritis, affects ~60% of lupus patients, and is considered an important cause of chronic renal failure (1, 3). The incidence and severity of renal involvement varies between patients with SLE, but seems to be ethnicity associated, as African-American, Asian or Hispanic patients are more likely to develop end-stage kidney disease compared to European-American lupus patients (4). Despite the availability of aggressive immunotherapies (i.e., CYC, MMF) (5), unfortunately associated with a plethora of side effects, chronic kidney disease (CKD) in SLE results in poor clinical outcome (6). Therefore, there is a constant and critical need to elucidate immune mechanism behind LN, in order to identify new and easily evaluated biomarkers facilitating precise diagnosis and patients monitoring. This would inevitably lead to the development of more effective and tailored therapies with better outcomes and fewer side effects. On the cellular level, B cells and T cells are still reckoned as the major culprits of immune dysregulation in SLE (7). Nonetheless, the innate immunity cells, especially dendritic cells (DCs), have moved to the fore, due to their natural ability to establish and maintain peripheral tolerance (2, 8).

Dendritic cells consist of heterogenous populations, specialized to interact with specific subsets of T cells upon stimulation with particular pathogen (9). The basic division distinguished classical or myieloid DCs and plasmacytoid DCs. mDCs can be further divided into type 1 - mDC1 and type 2 - mDC2 based on the surface marker expression [CD141+ (BDCA3+) and CD1c+ (BDCA1+), respectively], differential expression of key transcription factors and distinct functional features (9–11). CD1c+ fraction is a major population of human mDCs in body tissues, including lymph nodes, and body fluids (10, 12, 13). Due to higher easiness in acquisition, mCD2 are more widely used in laboratories (11) and clinics, including clinical studies on anti-tumor DC vaccination immunotherapies (13–15). Plasmacytoid DCs, the main source of IFNs after viral infections (7), express CD123, CD303 (BDCA-2), and/or CD304 (BDCA-4). Irrespective of subpopulation, DCs, while dysregulated, overshoot immune response, leading to imbalanced signaling and hyperactivation, thus having a strong impact on lupus pathogenesis (2, 16, 17). Therefore, it seems vital to unravel the molecular mechanisms involved in dendritic cell dysregulation in the course of SLE and its most common organ manifestation—LN. The emerging evidence shows that genetic and epigenetic factors are at play during the development of chronic kidney disease in lupus (18). Currently available data report the contribution of dendritic cells accumulated in nephritic kidney to intrarenal inflammation (8, 19). Unfortunately, the knowledge about the role of circulating peripheral dendritic cells and their epigenetic variations in the kidney injury, is still scarce.

In this study we analyzed the frequencies of two subpopulations of dendritic cells in peripheral blood in patients with renal manifestation of SLE. We also compared the extracellular co-stimulatory markers on dendritic cells from patients with age, sex and ethnicity matched healthy controls. We focused on gene expression patterns in lupus nephritis patients divided into groups based on the severity of chronic kidney disease. This division facilitated further epigenetic analyses comprising DNA methylation quantification and specific activation and repression histone marks, H4K4me3, and H3K27me3, respectively. The integration of gene expression pattern with DNA 5-mC content, followed with trimethylation of lysine's in histone H3 evaluation, expanded our knowledge about epigenetic landscape of dendritic cells in patients with LN. Moreover, our data enabled identification of factors that may serve as unique biomarkers in the diagnosis of lupus nephritis and monitoring of renal outcome in SLE patients.

## Materials and Methods

### Characteristics of the Patients

Blood samples were collected from systemic lupus erythematosus (SLE) patients (*n* = 51), and control subjects (healthy, with no history of autoimmune diseases, *n* = 19). SLE patients were under monitoring and treatment in the Department of Nephrology, Transplantology, and Internal Diseases, Medical University of Gdansk. All procedures were approved by Independent Bioethics Commission for Research in Gdansk (NKBBN/188/2012, issued: 22.05.2012) and informed written consent was obtained from all participants.

### Preparation of Peripheral Blood Mononuclear Cells (PBMC) and Flow Cytometry Analysis

The flow cytometry staining and analysis of DCs was preceded by PBMCs (peripheral blood mononuclear cells) isolation using histopaque density-gradient centrifugation (in accordance with the manufacturer's instructions—Sigma, USA). The phenotype and activation status of both subpopulations (myeloid and plasmacytoid) of DCs were examined. In order to obtain necessary data the following antibodies were used: Lin2- (CD3—clone MϕP9, CD14—clone SJ25C1, CD19—clone NCAM16.2, CD20—clone SK7, CD56—clone L27; Becton Dickinson, USA), anti-HLA-DR (clone LN3), CD11c (clone 3.9), CD1c (clone L161), CD123 (clone 6H6), CD303 (clone 201A) (eBioscience, Austria), and anti-CD80 (clone HB15e), CD83 (clone HB15) (Becton Dickinson, USA). The antibody cocktail—Lin2- was used to eliminate lymphocytes, monocytes, eosinophils, and neutrophils from the flow cytometry analysis. Peripheral blood dendritic cells could be then distinguished from other leukocytes by their lack of staining with Lin2. Based on surface markers expression the following subpopulations were identified: mDCs (HLA-DR, CD11c, CD1c), pDCs (HLA-DR, CD123, CD303), activated mDCs (HLA-DR, CD11c, CD80hi, CD83hi), inactive mDCs (HLA-DR, CD11c, CD80lo, CD83lo).

All flow cytometry analyses were performed on BD FACS LSRFortessa flow cytometer (BD USA) using BD FACSDiva software (BD Bioscience, USA). The acquisition gates were restricted to immune cell gates based on morphological characteristics, and at least 50,000 cells were acquired and analyzed. The results were expressed as a percentage of the studied cell population.

### Magnetic Isolation of mDCs and pDCs

Two populations of dendritic cells were isolated with two-step magnetic isolation starting from one sample of PBMCs. The isolation of myeloid DCs was performed by two magnetic separation sets according to the manufacturer's instructions—CD1c(BDCA-1)+ Dendritic Cell Isolation Kit (130-090-506, Miltenyi Biotec, USA) (13). First, B cells were depleted with CD19 MicroBeads. In the second step, CD1c (BDCA-1)+ mDCs in the CD19-depleted flow-through fraction were indirectly magnetically labeled with anti-CD1c (BDCA-1)-Biotin and Anti-Biotin MicroBeads. Upon separation, the labeled CD1c (BDCA-1)+ mDCs were retained within the column and eluted after removing the column from the magnetic field. The unlabeled cells retrieved from this step were subsequently used for further isolation of pDCs. Plasmacytoid dendritic cells were then separated with the means of CD304 (BDCA-4/Neuropilin-1) MicroBead Kit (130-090-532, Miltynei Biotec, USA). First the CD304+ cells were magnetically labeled with anti-CD304 MicroBeads. Then the cell suspension was loaded onto a column and placed in the magnetic field. After removing the column from the magnetic field, the magnetically retained CD304+ pDC were eluted as the positively selected cell fraction. The high >95% purity of mDCs and pDCs was determined by flow cytometry analysis. Purified mDC are contaminated with CD11c+ or CD1C+ cells, while purified pDC are contaminated with CD123+ or CD123-CD303- cells (Supplementary Figure 3). The insignificant number of the above-mentioned cells (<5%) has little impact on the parameters measured in this study.

### DC Gene Expression Analysis by Quantitative PCR

RNA was isolated using AllPrep DNA/RNA Mini Kit (Qiagen, Germany) according to the manufacturer's protocol. Quality and concentration of samples was evaluated using Epoch Spectrophotometer (Bio-Tek, USA). Total RNA was reverse transcribed into cDNA with iScript cDNA Kit (Bio-Rad, USA). For dendritic cells, the evaluation of 14 activity—associated transcripts was conducted with Locked Nucleic Acid probes from the Universal Probe Library—UPL (Roche GmbH Diagnostics, Germany). Real-Time PCR was performed using the LightCycler 480 Probe Master Kit (Roche Diagnostics GmbH, Mannheim, Germany) and Light Cycler^®^ 96 PCR System (Roche). Reactions were prepared in a total volume of 10 μl. The cycling conditions for UPL reactions were as follows: one cycle of 95°C for 10 min (initial denaturation), 45 cycles of 95°C for 10 s (denaturation) and 60°C for 30 s (annealing). Relative quantities of target genes were determined for unknown samples by the comparative threshold cycle (delta CT) method and normalized to HPRT1 quantities. Gene specific primers and probes sets (Roche Assay ID) used in the experiment are listed in Supplementary Table 1.

### DNA Methylation Quantification

DNA from mDCs and pDCs samples was isolated with AllPrep DNA/RNA Mini Kit (Qiagen, Germany), following the manufacturer's protocol. Quality and concentration of samples was evaluated using Epoch Spectrophotometer (Bio-Tek, USA). The ELISA-based Methylated DNA Quantification Kit (Colorimetric) (ab117128, Abcam, UK) was used to quantify global DNA methylation content in human dendritic cell subpopulations. The assay was performed in according to the manufacturer's instructions. Briefly, the 50 ng of total genomic from mDCs and pDCs DNA per reaction, was used for 5-methyl cytosine quantification, and relative DNA methylation was quantified using the positive control provided in the kit. The absorbance was read on a microplate reader at 450 nm (Victor X2, Perkin Elmer, USA). To determine the relative methylation status (percentage of 5-mC in total DNA) in our samples, the following formula was used:

$$5-mC\%\;=\left(\frac{\left(Sample\;OD-Negative\;Control\;OD\right)÷S}{Positive\;Control\;OD-Negative\;Control)\;\times 2^{*}\;÷P}\right)\times \;100$$

Where: S—amount of input sample DNA in ng; P—amount of input positive control in ng.

### Histone Extraction and Methylation Analysis

Total histone extracts from mDCs an pDCs, were isolated with Histone Extraction Kit (ab113476, Abcam, UK), according to the manufacturer's protocol. Next, histone samples were subjected to evaluation of global tri-methylation of histone H3K4 and H3K27 with two separate colorimetric assays: Histone H3 (tri-methyl K4) Quantification Kit (ab115056, Abcam, UK) and Histone H3 (tri-methyl K27) Quantification Kit (ab 115072, Abcam, UK). The absorbance was read on a microplate reader at 450nm (Victor X2, Perkin Elmer, USA). To calculate trimethylation of histone H3 at lysine 4 and 27, the following formula was used:

$$\begin{array}{l} trimethyaltion\;\%=\;\frac{Tested\;Sample\;OD-blank\;OD}{Control\;Sample\;OD-blank\;OD}\;\times 100\% \end{array}$$

### Statistical Methods

Statistical analysis was performed using GraphPad Prism 5.0 (GraphPad Software, USA) and Statistica 12.0 (StatSoft, Poland). Data are presented as medians and 25–75 quartile ranges unless otherwise stated. A comparison of parameters between all groups was made with the Kruskal-Wallis ANOVA test, and between two groups—with the Mann-Whitney *U*-test. Association between the groups were evaluated with Spearman Rank Correlation test. Results of the correlation analyses are presented as correlation coefficient. Statistical significance was accepted when *p* was ≤ 0.05. All analyses were performed with Statistica v.12 software, graphs were prepared either with Statistica v.12 or GraphPad Prism 5.

## Results

### Patient Cohort

Patients were enrolled in the study based on previous diagnosis [updated criteria of the American College of Rheumatology (20)] and positive results of following immunologic parameters: anti-nuclear antibodies (ANA) antibodies, anti-dsDNA antibodies, low serum levels of complement C3 and C4. Disease activity was measured with SLE Disease Activity Index [SLEDAI] (21, 22). Renal involvement was diagnosed on the basis of proteinuria, hematuria, and/or creatinine increase and eGFR values. Renal biopsy, performed on the majority of patients, in combination with laboratory results, allowed for the introduction of patient division according to CKD stage (Table 1). The aforementioned division, considering renal function, allowed to distinguish data associated strictly with lupus nephritis instead of considering them as assigned to systemic disease.

**Table 1:** Clinical and laboratory characteristics of individuals enrolled in the study.

	**Total LN *n* = 51**	**Mild LN (mild CKD—G1. G2) *N* = 35**	**Moderate LN (moderate CKD—G3a. G3b) *N* = 11**	**Severe LN (severe CKD—G4. G5) *N* = 5**	**Control group *n* = 19**
Age (years)	44.1 ± 12.3	43.4 ± 11.2	46.72 ± 17.12	42.6 ± 8.41	35.21 ± 8.59
Females %	82%	74%	100%	100%	73%
SLE duration (year)	16.77 ± 13.89	16.31 ± 16.13	17.36 ± 7.89	18.6 ±5.94	–
SLEDAI (score)	8.16 ± 7.87	7.23 ± 8.6	11.83 ± 6.03	6.6 ± 2.6	–
Symptoms (organs affected)^1^:					
Skin	41 (80%)	27 (77%)	10 (90%)	4 (80%)	–
Joints	43 (84%)	31 (88%)	0	1 (20%)	
Neurologic	5 (10%)	3 (8%)	10 (90%)	4 (80%)	
Hematologic	37 (%)	28 (80%)	6 (54%)	0	
Immunologic	51 (100%)	35 (100%)	11 (100%)	5 (100%)	
Antinuclear antibody	51 (100%)	53 (100%)	11 (100%)	5 (100%)	
Treatment^1^	46 (90%)	33 (94%)	10 (90%)	4 (80%)	–
Glucocorticoids Immunosuppressant	36 (71%)	26 (74%)	7 (63%)	3 (60%)	
Anti-malarial	19 (37%)	17 (48%)	2 (18%)	2 (20%)	
Anti—dsDNA (IU/ml)	260.19 ± 229.99	235.0 ± 215.42	307.94 ± 237.09	336.01 ± 327.29	N/A
ANA HEP2	2126.41 ± 751.79	2226.17 ± 702.72	2210 ± 829.29	1633.6 ± 878.11	N/A
Serum complement C3 (g/L)	0.99 ± 0.3	1.03 ± 0.31	0.88 ± 0.25	1.03 ± 0.4	N/A
Serum complement C4 (g/L)	0.16 ± 0.09	0.14 ± 0.07	0.16 ± 0.05	0.28 ± 0.18	N/A
Anti C1q antibodies (U/ml)	9.99 ± 18.49	8.88 ± 16.26	11.62 ± 19.59	14.1 ± 31.48	N/A
Hemoglobin (g/dL)	13.34 ± 1.82	13.85 ± 1.66^#^	12.5 ± 1.68	11.8 ± 1.87	N/A
Platelets (10^9^/L)	251.15 ± 85.03	258.94 ± 74.55	242.2 ± 81.42	220.8 ± 151.4	N/A
White blood cells (10^9^/L)	7.08 ± 2.74	6.58 ± 2.12	7.29 ± 1.87	9.78 ± 5.65	N/A
Neutrophils (10^9^/L)	4.68 ± 2.49	4.15 ± 1.63	4.7 ± 1.89	7.954 ± 5.14	N/A
Lymphocytes (10^9^/L)	1.6 ± 0.97	1.61 ± 1.04	1.84 ± 0.88	1.1 ± 0.27	N/A
Proteinuria (Protein reagent strip)	3.24 ± 15.87	0.69 ± 0.79	0.94 ± 0.85	0.54 ± 0.24	N/A
Daily proteinuria (g/day)	0.86 ± 1.32	0.77 ± 1.41	1.23 ± 1.26	0.54 ± 0.24	N/A
Erythrocyturia (cells/hpf)	3.11 ± 15.31	4.42 ± 18.17	0 ± 0	0 ± 0	N/A
Leukocyturia (cells/hpf)	4.25 ± 15.43	4.74 ± 18.24	3.4 ± 4.7	2.0 ± 3.46	N/A
C-reactive protein (mg/L)	4.91 ± 8.64	5.41 ± 10.01	3.0 ± 2.76	5.06 ± 4.13	N/A
Serum creatinine (mg/dL)	1.11 ± 0.58	0.84 ± 0.18*^#^	1.34 ± 0.21^∧^	2.72 ± 0.12	N/A
eGFR CKD-EPI (ml/min/1.73 m^2^)	68.96 ± 23.71	82.91 ± 11.54*^#^	45.8 ± 6.06^∧^	21.0 ± 4.08	N/A
Proteinuria	18 (35%)	11 (34%)	5 (45%)	2 (40%)	N/A
Renal insufficiency	15 (29%)	0	11 (100%)	5 (100%)	N/A

Data presented as mean ± SD.

1 Percentages do not equal 100% as patients may suffer from more than one symptom and can be given combinatory treatment. Statistically significant

* mild vs. moderate.

# mild vs. severe.

∧ *moderate vs. severe; p < 0.05*.

### Renal Involvement in SLE Affects Plasmacytoid DC Subpopulation

**Figure 1:**
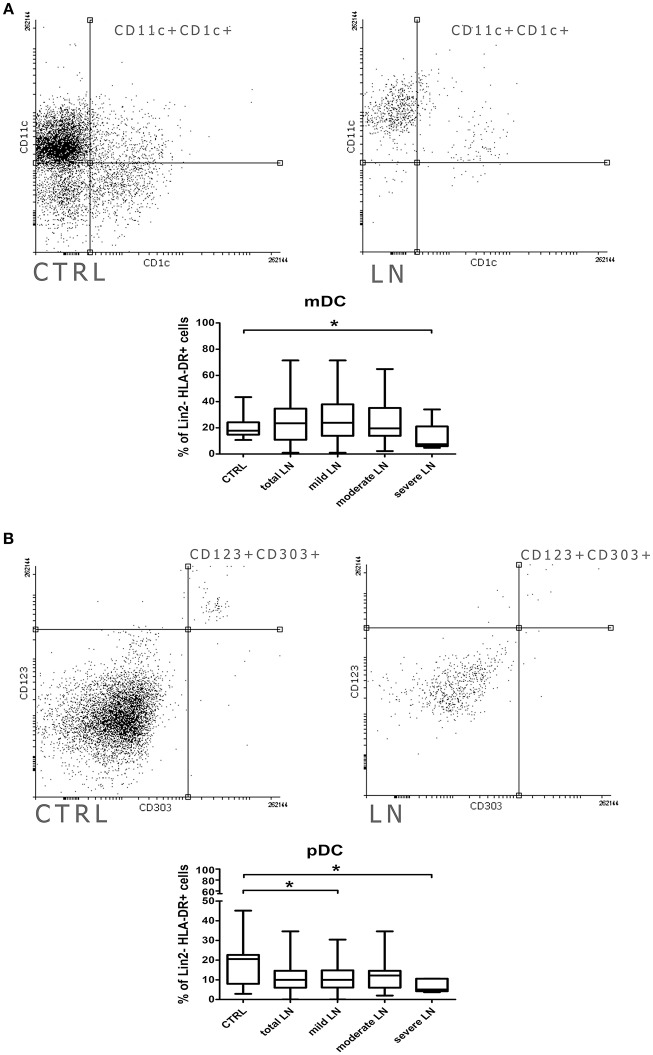
Alterations in dendritic cell subpopulations and their activity in lupus nephritic patients. PBMC isolated from blood samples collected from control volunteers and patients with LN were analyzed with flow cytometry in order to identify mDCs, pDCs and evaluate their frequencies. The samples were stained with monoclonal antibodies directed against: Lin2-, HLA-DR, CD1c, CD11c, CD123, CD303. Details of gaiting strategy are presented in Supplementary Figure 1. Data were collected with BD FACS LSRFortessa flow cytometer and analyzed with FACSDiva software. Graphic presentation of the results was prepared with Flowing Software v.2.5.1 (http://flowingsoftware.btk.fi/. Turku Centre for Biotechnology, University of Turku). **(A)** Representative FACS plots (gated on Lin-HLA-DC+ cells) showing peripheral mDCs from control person (left) and patient with LN (right). Graph show the percentage of mDCs (CD11c+CD1c+) in control group total number of LN patients and patients divided into groups based on the renal involvement (mild, moderate, severe LN). **(B)** Flow cytometry presentation of peripheral blood pDCs (CD123+ CD303+) gated on Lin-HLA-DR+ in control (left) and LN sample (right). The percentage differences in pDCs in all analyzed groups (control. total LN; mild, moderate, severe LN) are presented on the box and whiskers graph. Graph shows the statistically significant decrease in the percentage of pDCs in mild LN and severe LN groups compared to control. Data are shown as median and quartiles [25–75] (whiskers represent the minimum and maximum results) of the following number samples per group (CTRL *n* = 19, total LN *n* = 51, mild LN *n* = 35, moderate LN *n* = 11, severe LN *n* = 5). Significance was calculated in the relation to the control group. *p < 0.05 (Mann-Whitney *U*-Test).

The distribution of dendritic cell subsets in SLE patients was analyzed based on the expression of surface markers characteristic for either myeloid or plasmacytoid DCs. Cells expressing CD3, CD14, CD19, CD20, CD56 (Lineage cocktail—Lin 2-) were ruled out of the analysis, as representatives of other immune cell compartments. Identifying myeloid DCs (mDCs), we relied on expression of CD11c and CD1c and, thus, we observed comparable frequencies of this subpopulation in total LN patients and control group (Figure 1A). Only patients with severe LN displayed a statistically significant reduction in mDCs frequency. pDCs can be distinguished from other DCs by the expression of both CD123 and CD303. The flow cytometry analysis showed that the number of pDCs dropped significantly in the total LN patients population (Figure 1B). Once again, the severe LN group was characterized with the significant drop in pDCs number. Additionally, the mild LN group was characterized with statistically significant lower number of pDCs (Figure 1B).

### mDC Show Increased Costimulatory Molecule Expression at Early Stage of Renal Dysfunction in LN

**Figure 2:**
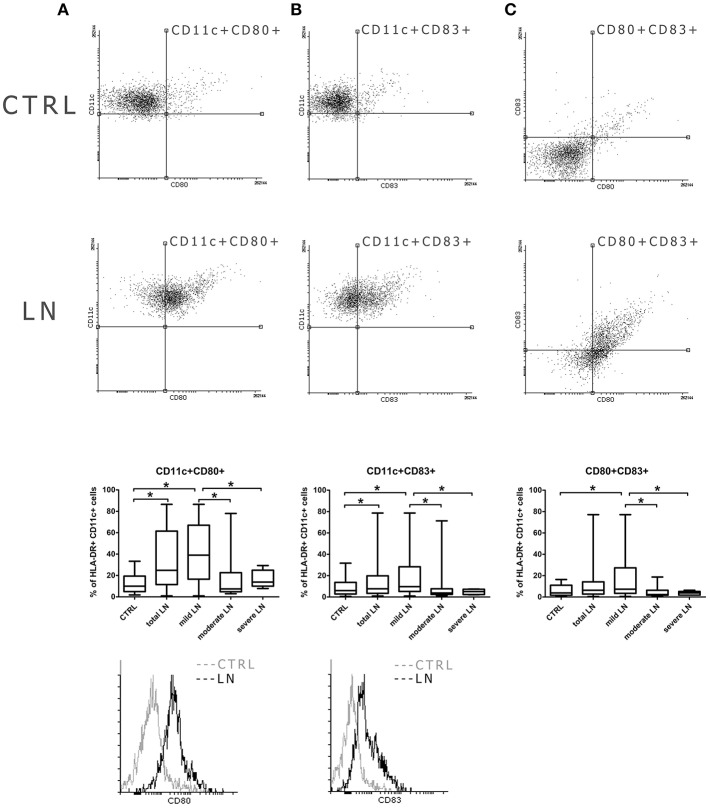
Increased co-stimulatory molecules expression in mDCs from LN patients. PBMC isolated from blood samples collected from control volunteers and patients with LN were analyzed with flow cytometry in order to evaluate their maturation and activity by measuring the expression of costimulatory molecules: CD80, CD83. The samples were stained with monoclonal antibodies directed against: HLA-DR, CD11c, CD80, CD83. Details of gaiting strategy are presented in Supplementary Figure 2. Data were collected with BD FACS LSRFortessa flow cytometer and analyzed with FACSDiva software. Graphic presentation of the results was prepared with Flowing Software v.2.5.1 (http://flowingsoftware.btk.fi/). Turku Centre for Biotechnology, University of Turku. **(A)** shows the analysis of CD11c+CD80+ mDCs. Representative dot plots show the frequencies of CD11c+CD80+ cells in control samples (top dot plot) and LN patient (bottom dot plot). Graph shows the percentage of CD11c+CD80+ gated on HLA-DR+CD11c+ mDCs in all analyzed groups with the emphasis on statistically significant changes. Overlaid histograms display the differences in CD80 expression between representative control sample (gray) and LN patient sample (black). **(B)** demonstrates the analysis of CD11c+CD83+ mDCs. Dot plots show the frequencies of CD11c+CD83+ cells in representative control samples (top dot plot) and LN patient (bottom dot plot). Graph shows changes in the percentage of CD11c+CD80+ between all analyzed groups, significant results are appropriately marked. Overlaid histograms display the differences in CD83 expression between illustrative control sample (gray) and LN patient sample (black). **(C)** presents the analysis of double positive mDCs (CD80+CD83+). Dot plots show the number of cells expressing both maturation/co-stimulatory markers in both control samples (top dot plot) and LN patient (bottom dot plot). Graph exhibits differences in the number of double positive mDCs changes between control group and LN groups (total LN; mild. moderate and severe LN) statistical significance is included. **(A–C)** Data are shown as median and quartiles [25–75] (whiskers represent the minimum and maximum results) of the following number samples per group (CTRL *n* = 19, total LN *n* = 51, mild LN *n* = 35, moderate LN *n* = 11, severe LN *n* = 5). Significance was calculated in the relation to the control group. *p < 0.05 (Mann-Whitney *U*-Test).

Even though the frequencies of mDCs were not significantly altered in patients with lupus, we observed some changes in their activation features/attributes. To evaluate the activation and maturation of analyzed DC subpopulations we measured the expression of two surface proteins (CD80, CD83), referred as activation and antigen presentation capability markers/positive co-stimulatory molecules. We perceived a remarkable increase of mDCs expressing CD80 in patients suffering from lupus compared to control subjects (*p* = 0.002) (Figure 2A). Next, we conducted a similar analysis taking into consideration CKD stages and we observed the increase of CD11c+CD80+ DCs number in mild LN patients compared to control group. This increment also reached significance when the comparison was made between mild LN and both moderate and severe LN groups, underlying the role of DCs activity at early stages of kidney disease. Similar observation was made for the CD11c+CD83+ mDCs (Figure 2B), as mild LN group demonstrated its significant predominance in the frequency of these activated DCs over other tested groups: both moderate and severe LN along with healthy control. Therefore, based on above mentioned data, we could have expected subsequent results for double positive activated mDCs—CD80+CD83+ (Figure 2C). Indeed, the most notable increase was once again distinguishing for mild LN against the remaining groups. Hence, we may state that the most active mDC phase may occur concomitantly with early stages of renal dysfunction.

### Differential Expression of Interferon Regulatory Factors in mDCs and pDCs From LN Patients

In order to evaluate the expression profile of the two analyzed DC subpopulations, the cells were magnetically separated, based on the characteristic surface markers (details can be found in the material and method section). Purified mDCs and pDCs gene expression patterns were evaluated by TaqMan QPCR analysis. We focused on a selected set of genes associated with the generation, functioning and signaling of each DCs subpopulation (Supplementary Table 1). To clarify the acquired data, analyzed genes were divided into categories according to their role in the above listed processes. A gene within each category was considered differentially expressed if at least a 2.0-fold difference was observed between SLE groups and controls.

**Figure 3:**
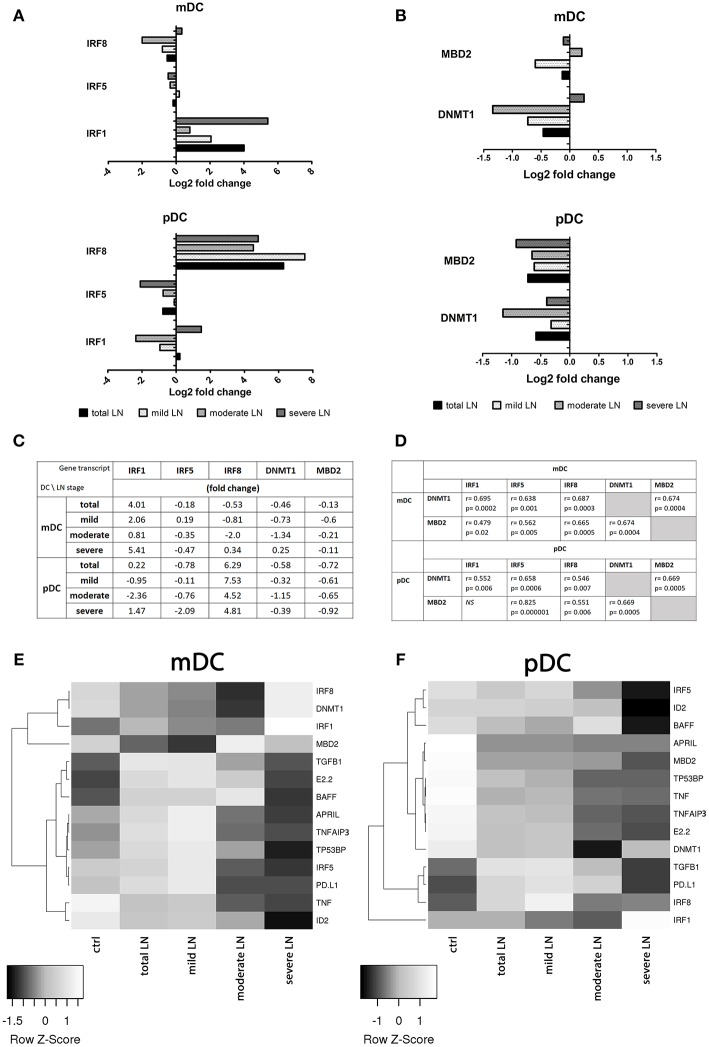
Comparison of gene expression profile in healthy and circulating lupus nephritis-affected mDCs and pDCs. To evaluate the transcription profile of mDC and pDCs magnetically isolated DC subpopulations were subjected to QPCR analysis of a set of genes associated with their activity and function. **(A)** Gene expression fold change differences between LN patients with various stages of CKD: mild, moderate, and severe. Graphs represent changes in IRF genes transcription, showing significant differences in IRF activation pattern between DCs subpopulations. **(B)** Differences in the expression of epigenetic regulating genes: DNMT1 and MBD2 in both mDCs and pDCs, represented as fold change (log2 based). **(C)** Representative values of fold differences of selected genes in both mDCs and pDCs detected between control and studied groups. **(D)** Suggestive associations between epigenetic-related genes (DNMT1, MBD2) and interferon regulatory factors (1,5, and 8) in DC subtypes. Tables presents correlation coefficient values of Spearman Ran Correlation test and *p*-values for each association. **(E)** Rowscaled clustering heatmap according to Pearson correlation of differentially expressed genes in mDCs between control volunteers and LN samples (left). **(F)** Heatmap of pDCs gene expression comparison between the control and LN groups (right). **(A–F)** Relative genes expression was calculated using delta CT method and normalized to HPRT1 quantities. **(E,F)** Heatmap color-coding: white (upregulated), black (downregulated; data are presented as median value from each of the analyzed group. **(A–E)** Data on the graphs are shown as median and quartiles [25–75] (whiskers represent the minimum and maximum results) of the following number samples per group (CTRL *n* = 19, total LN *n* = 51, mild LN *n* = 35, moderate LN *n* = 11, severe LN *n* = 5). Significance was calculated in the relation to the control group. *p < 0.05 (Mann-Whitney *U*-Test).

The analysis of the expression patterns of the genes involved in the transcription regulation revealed that all three analyzed IRF (1, 5, and 8) genes were differentially transcribed dependent on CKD stage (Figure 3A). mDCs from mild, moderate and severe LN patients showed increased expression of IRF1. Hence, the upregulation in IRF1 transcription in the severe LN group revealed significant augmentation in comparison to both control individuals and patients with mild kidney involvement. In pDCs the transcription of this factor was also visibly altered but did not reach the significance. Only patients with severe LN revealed this transcript upregulation, whereas mild LN and moderate LN groups displayed downregulation trend. IRF5, known as SLE enhancer, was rather downregulated in both mDCs and pDCs in the majority of patients with renal involvement. Furthermore, only pDCs from severe LN group exhibited significant decline in expression pattern of IRF5 compared to the control group and mild LN patients. IRF8, as the only one, showed a differential expression pattern between total SLE group and control individuals. The IRF8, a transcription factor from interferon-regulatory factor (IRF) proteins family, was downregulated in mDCs from SLE patients. Interestingly, the second subpopulation of DCs—plasmacytoid—revealed remarkable upregulation of this gene. Moreover, separate analysis of DCs subpopulations, revealed that only in mDCs from patients with severe nephritis, the level of IRF8 transcript was increased, whereas two remaining patient groups (mild and moderate LN), showed inverted tendency. The most notable, but not statistically significant drop, was recorded for moderate CDK patients. On the other hand, expression differences noted for severe LN were significant in comparison to mild LN group and healthy individuals. Considering pDCs, we observed the most remarkable upregulation of IRF8 regardless the patient group. In mild LN, the transcript level of IRF8 was 7.5-fold higher than in control samples, while for moderate LN the fold reached the value of 4.5, and for severe LN−4.8. Still the two latter groups presented significantly lower values compared to mild LN patients. ID2 and E2-2, genes from transcription regulation category, were not differentially transcribed between studied groups (fold <1.0).

### DNMT1 and MBD2 Transcription in LN Is Associated With IRFs Levels

As to genes associated with epigenetic status of the cell, we observed either moderate or insignificant alterations in mRNA levels (fold <2.0). DNMT1, responsible for the maintenance of DNA methylation pattern, and MBD2, a DNA demethylase, were downregulated in mDCs from total SLE patients (Figures 3B,C). Interestingly, the group of patients with moderate LN showed downregulation of DNMT1 and a slight upregulation of MBD2 in mDCs (Figures 3B–D). Mild LN group revealed the decline tendency for both genes associated with the epigenetic status of mDCs. Patients with severe kidney disease exhibited a slight increase in DNMT1 transcription, accompanied with small downregulation of MBD2 (Figures 3B–D). pDCs from total SLE patients, regardless of kidney involvement, transcribed DNMT1, and MBD2 at lower levels compared to control. The one and only distinct result was obtained in moderate LN patients considering distinctive downregulated transcription of both DNMT1 and MBD2 in pDCs (Figures 3B,C,F). Other groups of LN patients, including the mild and severe groups, were also characterized with the downregulation of DNMT1 and MBD2 transcription in pDCs. These two epigenetic associated transcripts appear to be significantly associated with the transcription factors from IRF family. Medium and strong correlations were identified between the examined IRFs (1, 5, and 8) and DNMT1 and MBD2 in both DCs subpopulations (correlation coefficients are presented in Figure 3D).

### Altered Gene Expression Profiles in mDCs and pDCs in SLE

Most genes implicated in regulation of immune response in mDCs were differentially transcribed, with visible upregulation in mild LN group (Figure 3E). BAFF, TNFAIP3, and TP53BP1 were upregulated in mDCs isolated from patients with mild LN. pDC from the same groups of patients revealed a distinct expression pattern for genes in control of immune reactivity (Figure 3F). Genes from this category (APRIL, TNFAIP3, and TP53BP1) were evidently downregulated in pDCs from mild LN patients compared to control. Only fold-change of BAFF expression in pDCs did not reach the set threshold, showing minimal increase in transcription level in moderate LN group. The immune response associated genes—TNF and TGFB1—representatives of pro- and anti-inflammatory immune reaction cytokines respectively, showed opposite expression tendencies. While TGFB1 was significantly upregulated in both mDCs and pDCs, TNF was downregulated, either moderately in mDCs or noticeably in pDCs.

### DNA Hipermethylation in Severe Chronic Kidney Disease in SLE

**Figure 4:**
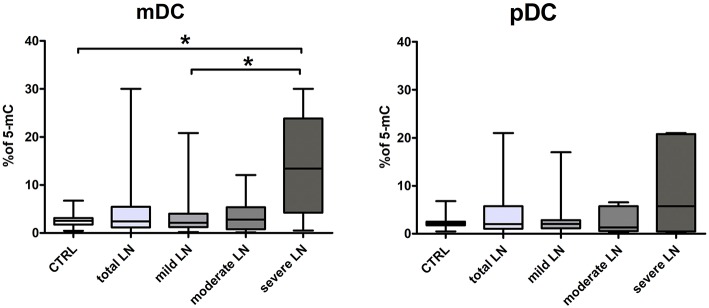
Increased percentage of 5-mC in DCs DNA from LN patients. The analysis of global DNA methylation in mDCs and pDCs was performed utilizing colorimetric quantification kit. Graphs present changes in the percentage of methylated cytosine in mDC (left) and pDCs (right) in control samples and LN patients groups. Data on the graphs are shown as median and quartiles [25–75] (whiskers represent the minimum and maximum results) of the following number samples per group (CTRL *n* = 19, total LN *n* = 51, mild LN *n* = 35, moderate LN *n* = 11, severe LN *n* = 5). Significance was calculated in the relation to the control group. *p < 0.05 (Mann-Whitney *U*-Test).

We performed an analysis of global DNA methylation in mDCs and pDCs from a complete set of SLE patients and normal controls using a colorimetric quantification kit. The median percentage of methylated DNA sequences in control group was 2.54 for mDCs and 2.1 for pDCs. Data obtained for the total LN group did not reach significance compared to control regardless DC subpopulation. Though, we could observe slight increasing tendencies in LN patients (Figure 4). Having focused on kidney involvement in the course of lupus, we uncovered significant alterations in global percentage of 5-Methylcytosine. The acquired data revealed that mDCs from patients with severe LN in lupus were characterized with a statistically significant increase in percentage of 5-mC compared to healthy controls and mild LN group (Figure 4). Comparable increase tendency was observed in severe LN pDCs, though no statistical significance was reported (Figure 4). Moreover, weak and moderate positive associations were detected between the proportion of 5-mC in mDCs and disease duration (*r* = 0.35, *p* < 0.05) and levels IRF1 transcript in mDCs (*r* = 0.51, *p* < 0.05), respectively. Moderate negative correlation was found between 5-mC in mDCs and IRF5 expression in mDCs. The DNA methylation in pDCs also corresponded with other analyzed features, i.e., with ANA HEp2 (*r* = −0.39, *p* < 0.05), IL-6 and IL-8 (*r* = −0.35 and *r* = 0.34, respectively, *p* < 0.05), or level H3K4me3 in pDCs (*r* = 0.39, *p* < 0.05).

### Correlation of Histone Modifications and Gene Expression

**Figure 5:**
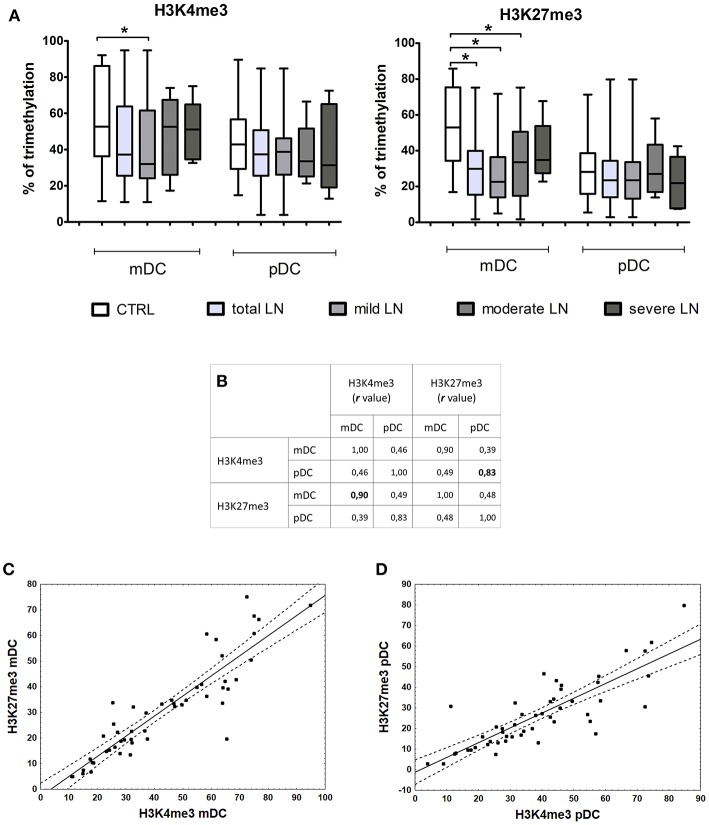
Changes in histone marks in patients with LN. Histone extracts obtained from magnetically separated mDCs and pDC were subjected to colorimetric evaluation of H3K4me3 and H3K27me3. **(A)** Graphs represent the percentage of trimethylation of H3K4 (left) and H3K27 (right) in both DC subpopulations. The decline in epigenetic marks is noticeable between control samples and LN patients groups, particularly in mDCs. **(B)** Spearman Rank Correlation test revealed high positive associations between H3K4me3 and H3K27me3 in each DC subtype. Table shows correlation coefficient values statistically significant associations are in bold. **(C)** Correlation between H3K4me3 and H3K27me3 in mDCs, linear regression with a 95% confidence interval. **(D)** Correlation between H3K4me3 and H3K27me3 in pDCs, linear regression with a 95% confidence interval. Data on the graphs are shown as median and quartiles [25–75] (whiskers represent the minimum and maximum results) of the following number samples per group (CTRL *n* = 19, total LN *n* = 51, mild LN *n* = 35, moderate LN *n* = 11, severe LN *n* = 5). Significance was calculated in the relation to the control group. *p < 0.05 (Mann-Whitney *U*-Test).

Epigenetic induction/repression of transcription may be mediated not only through DNA methylation, but also through histone modifications. Methylation of lysine in histone H3 can be connected either with gene activation (H3K4me3) or gene repression (H3K27me3). The analysis of lysine 4 and 27 in histone H3 revealed notable differences between the percentage of H3K4me3 (not significant) and H3K27me3 (significant, *p* = 0.0003) in mDCs from total LN group compared to controls (Figure 5A). pDCs showed no remarkable alterations between groups, since the observed data fluctuations were in the range of control values. Contrary to this, mDCs exhibited a visible decline in the level of lysine 4 trimethylation in mild LN group in comparison to healthy volunteers (Figure 5A). Another noteworthy result was detected for H3K27me3—repressive chromatin mark, since statistically significant decrease in trimethylation was observed in total LN group. Regarding kidney involvement, especially with mild and moderate CKD, revealed significantly decreased levels of H3K27me3 in mDCs (*p* = 0.0003 and *p* = 0.035, respectively). The obtained data also revealed several suggestive associations for both activation and repression H3 methylation markers in mDCs. The expression of IRF1 in mDCs showed moderate positive correlation with both H3 trimethylated lysines (K4, K27). Other positive associations with H3K4me3 were found for IRF8, APRIL, and TP53BP1 transcripts in mDCs. MBD2 expression turned out to be positively linked to both chromatin marks: H3K4me3 in mDCs and H3K27me3 intensity in pDCs. Moreover, high positive correlation rates were detected between the intensities of both methylation marks (Figure 5B). The hallmark of activity—H3K4me3—showed very high association with repressive H3K27me3 in mDCs (*r* = 0.9, *p* < 0.05; Figure 5C). The same situation was observed in pDCs (Figure 5D), as both H3K4me3 and H3K27me3 were positively (*r* = 0.83, *p* < 0.05) highly associated in this APC subpopulation.

## Discussion

Recent years have introduced new cellular players to autoimmunity, thus providing new directions toward understanding the pathogenesis of SLE. Dendritic cell, originally identified as professional antigen presenting cells, proved to be vital in induction and maintenance of immune tolerance (23). These cells, regardless the subpopulation, while dysregulated are anticipated to be responsible for the tolerance breakdown resulting in autoaggression (24). Abnormalities in circulating DCs function have been linked to the systemic manifestation of lupus, throughout the interplay with both B cells (25) and T cells (26). Our previous study revealed that molecular alterations in mDCs (CD1c+) and pDCs from lupus patients significantly influence the hyperactivated B cells, mainly through secretory and expression activity [unpublished data]. Nonetheless, little is known about the role of circulating peripheral subpopulations of DCs in the most deleterious organ manifestation of SLE—lupus nephritis. The majority of published studies were focused on local inflammation environment in the kidney, including IC formation and deposition in glomeruli, expression of proinflammatory cytokines promoting the influx of immune cells (12, 19, 27, 28). Moreover, pDCs are widely known to preferably migrate from circulation into inflamed tissues (29, 30), but the role of mDCs (CD1c+) in kidney insufficiency remains unclear, although there are some data showing disturbed distribution of CD1c+ DCs in renal biopsies of nephritic patients of various origin (12).

The major goal of presented data was to attract the scientific environment attention to the role of DCs in the development of nephritis in SLE. Our observation confirms the hypothesis according to which pDC tend to infiltrate affected kidneys, therefore limiting their number in the circulation. We are the first to prove that, even reduced in number, circulating pDCs might be of vital importance in the diagnosis and monitoring of chronic kidney disease. In presented study all patients with LN were characterized with significantly lower numbers of pDC in the peripheral blood, furthermore patients with severe CKD showed the most limited percentage of pDCs. Hence, it may suggest that the decreasing population of pDCs could be a predictor of progressing renal failure. On the contrary, even though mDCs number did not show any alterations in our study, we could observe an increase in their co-stimulatory potential. mDCs with higher expression of surface CD80 and CD83 were more prevalent in patients with mild CKD. Thus, we can hypothesize that the most active mDC phase occurs concomitantly with early stages of renal dysfunction. Both peripheral DC subpopulations revealed specific changes, that makes them suitable alternative as diagnostic tool omitting invasive biopsy procedure.

All patients enrolled in the study were given a standard treatment, as a routine maintenance provided by the clinic. This may arise a question whether the therapeutic regimen could affect the frequencies of mDCs and pDCs. The majority of our patients (90%) were subjected to standard SLE/LN treatment based on glucocortycosteroids, that are known to attenuate immune system reactivity. Steroids in combination with conventional immunosuppressive agents are also widely used in order to suppress immune complex-mediated inflammation and to induce immune quiescence (31). Approximately 70% of LN patients in this study were treated with immunosupressants (i.e., MMF). According to literature, the currently available SLE treatment, both conventional and based on biological agents, is targeted at specific subsets of adaptive immune cells, mainly B cells (32, 33) T cells (31, 34, 35). In therapeutic aspects, dendritic cells are rather reckoned as tools (36) not targets, i.e., (DC)-based vaccination (37). Therefore, we do not expect that the frequencies of analyzed dendritic cells were significantly negatively influenced by the applied treatment. Still, it seems worthwhile to elucidate the impact of immunotherapeutic milieu on the activity of isolated dendritic cells.

The analysis of interferon regulatory factors—IRFs- transcripts levels, confirmed previous statements about their role in the pathogenesis of SLE (38). Our data imply that IRFs also contribute to LN, with preferable pattern of expression dependent on DC subpopulation. Transcription factor—IRF1—controls the ability of DC to promote Th1 differentiation by creating propitious cytokine milieu, abundant in IL12 and IL18 (39). Animal studies showed that the expression of IRF was highly induced in glomeruli of mice with IFNα-induced LN (38, 40, 41). The upregulation of IRF1 expression, noted for LN patients, was in line with our observation of preferential maturation of mDCs, particularly in mild LN group. These exceptionally high levels of IRF1 mRNA in circulating mDCs from severe LN patients, observed in our study, could be predictive of both renal treatment response and insufficiency progression in LN.

pDCs gene expression pattern, detected in our study, was dominated with the outstanding upregulation of IRF8 expression. IRF8 is widely known as one of the transcription factors coordinating DC lineage commitment (9, 42) and its alleles have been associated with SLE and MS (43). Little is known about IRF8 role in autoimmune nephritis. Few studies, mainly conducted in animal models, managed to prove that IRF8-deficiency could reduce kidney disease (44, 45). Our results, showing the remarkable increase in IRF8 transcription in pDCs from patients with kidney manifestation of SLE, pave a new path for understanding the role of IRF8 in the pathogenesis of SLE and LN.

IRF5 is associated with SLE through a risk haplotype (46, 47), not by changes in its transcription levels. Therefore, low expression of this factor in our study was not unexpected. But the presented pattern showed significant decline in severe LN group, suggesting IRF5 diminishing role with the perpetuation of renal insufficiency.

The suggestive associations detected between IRFs and epigenetic related factors: DNMT1 and MBD2, encouraged us to explore epigenetic aspects of lupus nephritis, as not only genetic changes may contribute to disease progress. We observed an interesting tendency of DNMT1 and MBD2 downregulation in mDCs and pDCs, regardless the disease state. The upregulation was noted only for mDCs from moderate and severe LN. These data may suggest that epigenetic changes might be controlled by other pathways and mechanisms (2, 48, 49), with inconsiderable engagement of the above mentioned factors. DNA demethylation, relaxing chromatin structure, facilitates the access of transcription factors thus inducing transcriptions of genes. SLE is widely linked to hypomethylation DNA signature (50, 51) and the overexpression of several disease aggravating genes (1). Arguing against that hypothesis are our results of global DNA methylation from peripheral blood mDCs and pDCs. DNA from both DC subpopulations was not characterized with decreased levels of 5-mC in majority of the patients. The remarkable exception was the group of patients with severe LN. The DNA hypermethylation observed in this group can be treated as an outstanding feature, worth being considered as chronic kidney disease progression biomarker. On the other hand, the group of patients with advanced renal dysfunction were characterized with the most noticeable downregulation of analyzed gene transcripts. This low transcriptional activity of DCs might be associated with general attenuation of immune system, as end stage renal disease (ESRD) often results in increased susceptibility to infections, impaired response to vaccinations and lymphopenia (52–54). Nevertheless, this phenomenon requires further investigation to identify the regions with increased methylation status, thereby providing an association between gene expression changes and development of autoimmune kidney end stage disease.

The activity and function of peripheral blood dendritic cells can be also regulated by epigenetic histone modifications. Gene-specific regulation on the chromatin level may include nucleosome remodeling and covalent histone modifications (55), i.e., methylation of N-terminal tail (56). In our study we focused on two specific modifications of histone H3, regarded as enhancer and repressor epigenetic marks of transcription, H3K4me3, and H3K27me3, respectively (57). It has been proven that epigenetic modifications on the histone level are involved in the plasticity of human DCs in different environments. Moreover, the changes in genes regulation do not always depend on the increased/decreased level of either H3K4me3 or H3K27me3, but rather on alterations of both epigenetic marks (58). It has been reported that in immune cells many active genes do have increased H3K4me3 marks and repressed genes are characterized with modified H3K27me3 marks (57, 58). Having focused on dendritic cells, we could add some new information to previously published data. The most significant changes in histone marks affected mDCs, revealing decreased H3K4me3 and H3K27me3 marks in patients compared to unrelated healthy individuals. Obtained results were in line with findings, that induced genes do not always rely on increased levels of H3K4me3, and the downregulation of genes do not have to result from the presence of H3K27me3. Additionally, we observed strong positive correlation between these two modifications of H3 in both analyzed DC subpopulations. The coexistence of these two conflicting marks, initially described as an element of lineage specific embryonic stem cells differentiation system (59), is suggested to provide transcriptional plasticity (60). Studies, utilizing reChIP-seq, showed that hypomethylated CpG-rich promoters were either H3K4me3-only and transcriptionally active or bivalent and transcriptionally repressed. This mechanism is believed to protect against excessive deamination in promoter regions (61). These findings stress the importance of H3K4me3/H3K27me3 bivalency in dysregulation of immune cells activity in autoimmune processes. We made the first step on this path revealing altered presence of epigenetic marks in patients with SLE. Moreover, a global decrease of these two methylation marks was characteristic for patients with renal involvement. Further studies, exploring promoter and enhancers regions enriched in H3K4me3 and H3K27me3 marks, would make us closer to tailored diagnosis and monitoring of kidney disease in SLE.

Herein, we presented a novel and vital data regarding dendritic cell contribution to the pathogenesis and propagation of lupus nephritis. The alteration in mDCs and pDCs accessible in peripheral blood, DCs activity, genetic, and epigenetic landscape, could be critical in autoimmune diseases, although not yet fully understood and appreciated. Based on the presented data, we can support the statement that visible changes in the flow cytometry evaluation of circuiting pDCs frequencies are associated with progression of kidney damage. Therefore, the limited number of pDCs in peripheral blood could be a practical and convenient method of CKD diagnosis and monitoring. Moreover, our results imply suggestive new biomarkers of LN pathogenesis and development, emphasizing the role of genetics and epigenetics in autoimmunity. The initial increase in upregulation of IRF1 expression, coinciding with the initial mild kidney chronic disease, might be considered a prognostic factor of renal scarcity. On the other hand, the vast acceleration of IRF1 transcription in mDCs from severe LN patients, could easily substitute invasive biopsy-based method of kidney insufficiency evaluation. Furthermore, the decline in epigenetic H3 histone marks (H3K4me3, H3K27me3) is worth mentioning as possible biomarker of immune system dysregulation in LN, thus prognosis for kidney dysfunction progression. All the above-mentioned observations, putative new biomarkers of LN pathogenesis, progression, and treatment response, require further insight. However, they do already constitute an appealing alternative for currently used invasive methods, i.e., biopsies, required in nephritic patients monitoring.

## Data Availability

The datasets generated for this study are available on request to the corresponding author.

## Author Contributions

AW designed the study, obtained funding, analyzed the clinical and experimental data, and wrote the manuscript. MK and BB-P collected clinical material. AW, MK, and MP analyzed the clinical and experimental data. BB-P and MD-Ś analyzed clinical data. AW, MD-Ś, and MP revised the manuscript. All the authors approved the final version of the manuscript.

### Conflict of Interest Statement

The authors declare that the research was conducted in the absence of any commercial or financial relationships that could be construed as a potential conflict of interest.
