# Supplementary material for: Transcriptomic and Epigenetic Alterations in Dendritic Cells Correspond With Chronic Kidney Disease in Lupus Nephritis
Source: Front Immunol. 2019 Aug 27;10:2026. doi: 10.3389/fimmu.2019.02026 (PMC6718474; doi:10.3389/fimmu.2019.02026)
Supplement: Supplementary file 1 [file Data_Sheet_1.pdf]

Supplementary material

Table of contents:

1. Supplementary Table 1 - Gene specific primers and probes sets (Roche Assay ID) used in the experiment
2. Supplementary Figure 1. Gating strategy for mDC and pDCs from human PBMC
3. Supplementary Figure 2. Gating strategy for activated mDC from human PBMC.
4. Supplementary Figure 3. Purity of magnetically separated DCs subpopulations.

Supplementary Table 1. Gene specific primers and probes sets (Roche Assay ID) used in the experiment

Gene symbol	Assay ID	Name [alias]	Function
HPRT1	145173	hypoxanthine guanine phosphoribosyltransferase	housekeeping gene; transferase in purine salvage pathway
PD-L1	104030	Programmed cell death 1 ligand 1 [CD274]	Surface receptor, key role in the inhibition of immune response
DNMT1	102318	DNA methyltransferase 1	transfers methyl groups to cytosine nucleotides of genomic DNA, maintaining methylation patterns following DNA replication
ID2	102019	inhibitor of DNA binding 2	Transcriptional regulator, inhibitor of differentiation
IRF1	144798	interferon regulatory factor 1	transcriptional regulator, activator of genes involved in both innate and acquired immune responses
IRF5	103573	interferon regulatory factor 5	transcription factors, immune system activator, known to enhance SLE
IRF8	116597	interferon regulatory factor 8	transcription factor, regulator of B cells differentiation, SLE susceptibility gene, required for DCs development
MBD2	115074	methyl-CpG binding domain protein 2	nuclear protein, transcription repressor of methylated gene promoters, role in gene silencing
E2-2	112749	transcription factor 4 [TCF4]	Transcription factor, essential to maintain pDCs phenotype
TGFB1	101210	transforming growth factor beta 1	Multifunctional protein, regulates cell proliferation, differentiation and growth
TNF	147880	Tumor necrosis factor [TNFalpha]	multifunctional proinflammatory cytokine, cell proliferation,

			differentiation, apoptosis, lipid metabolism, and coagulation
TNFAIP3	111227	TNF alpha induced protein 3 [A20]	Negative regulator of immune response, inhibit NF-kappa B activation, involved in the cytokine-mediated immune and inflammatory responses
TNFSF13	111225	TNF superfamily member 13 [APRIL]	Proliferation inducing ligand, vital for survival and differentiation of B cells
TNFSF13B	104221	TNF superfamily member 13b, [BAFF]	B cell activating factor, vital for survival and differentiation of B cells
TP53BP1	114211	tumor protein p53 binding protein 1 [p202]	plays multiple roles in the DNA damage response, overexpression in DC leads to hyperactivation and prolonged lifespan,

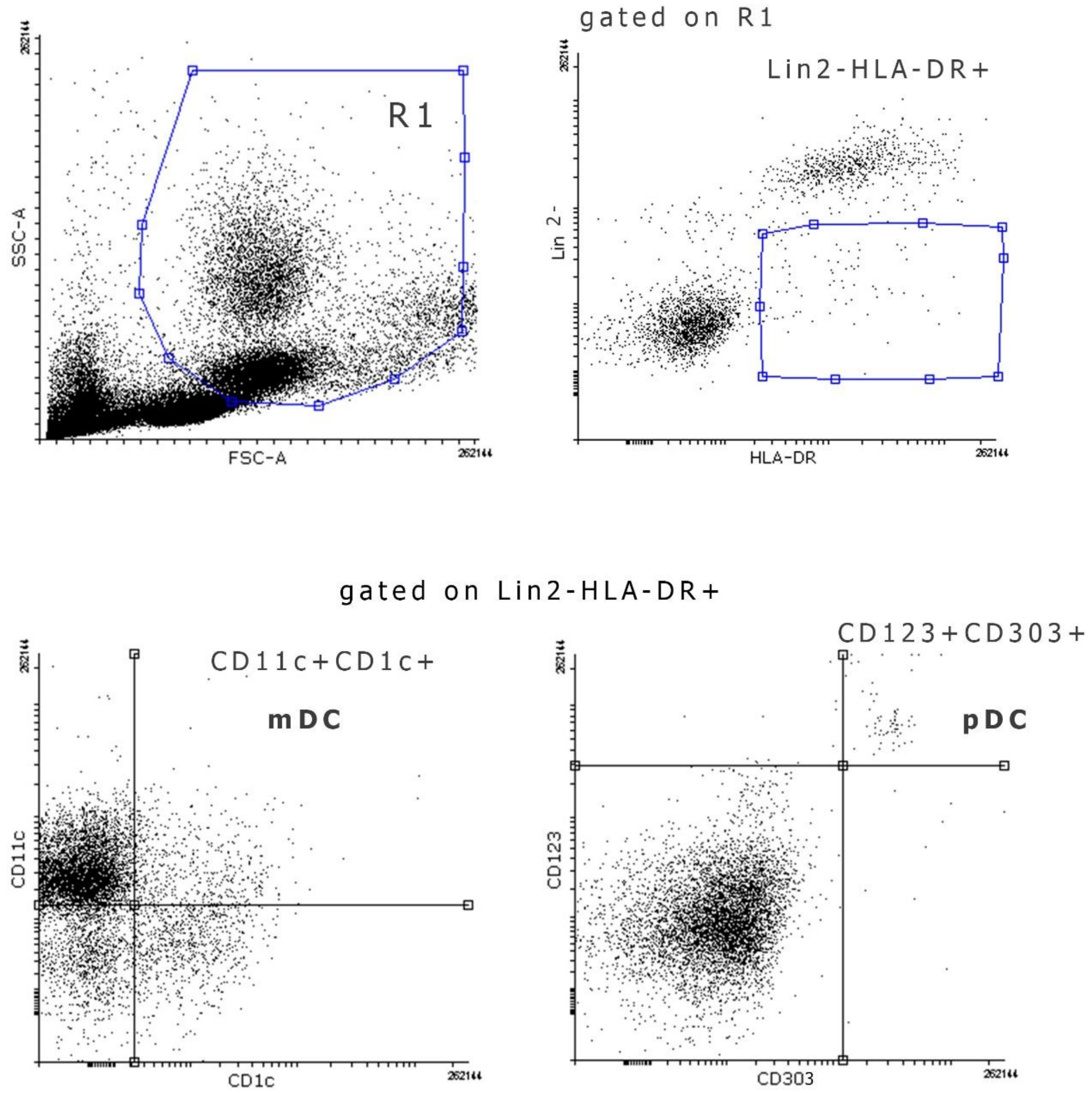

Supplementary Figure 1. Gating strategy for mDC and pDCs from human PBMC. Whole human PBMC were initially visualised based on FCS and SSC. R1 gate, excluded debris and lymphocytes, and allowed next gating based on the negative expression of Lin2- (cocktail of antibodies against: CD3, CD14, CD19, CD20, CD56) and positive expression of HLA-DR+. The subsequent gating on CD11c+ CD1c+ and CD123+CD303+ selectively identified mDCs and pDCs, respectively.

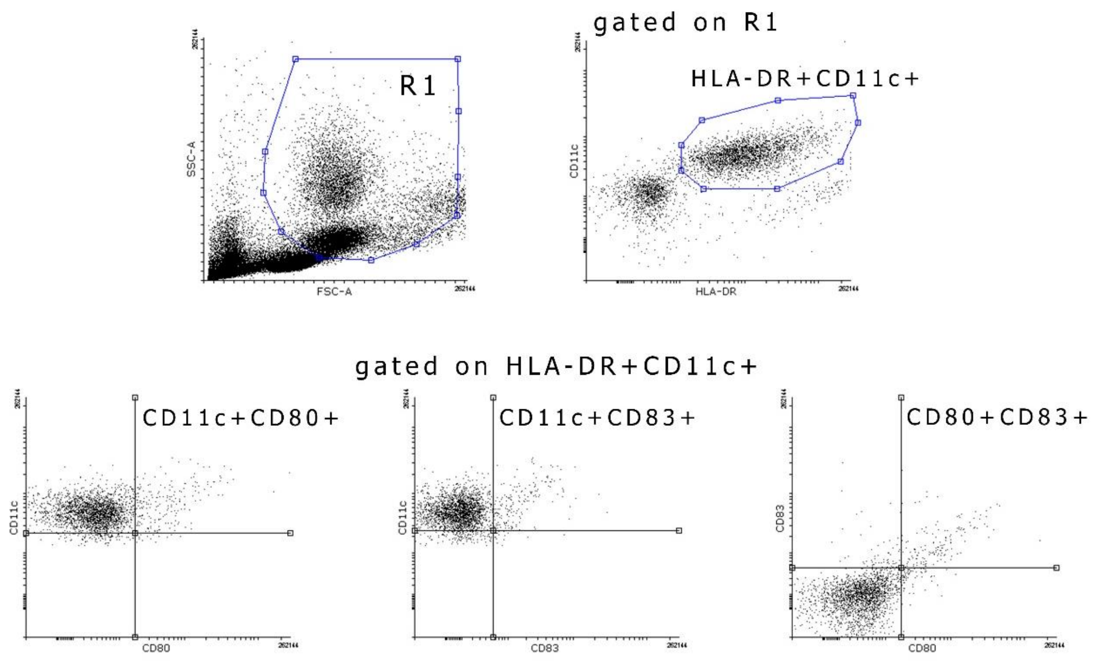

Supplementary Figure 2. Gating strategy for activated mDC from human PBMC. Whole human PBMC were initially visualised based on FCS and SSC. R1 gate, excluded debris and lymphocytes, and allowed next gating based on the positive expression ofHLA-DR+ and CD11c+. The subsequent gating on CD11c+ CD80+, CD11c+ CD83+ and CD80+ CD83+ identified mDCs with increased expression of co-stimulatory molecules.

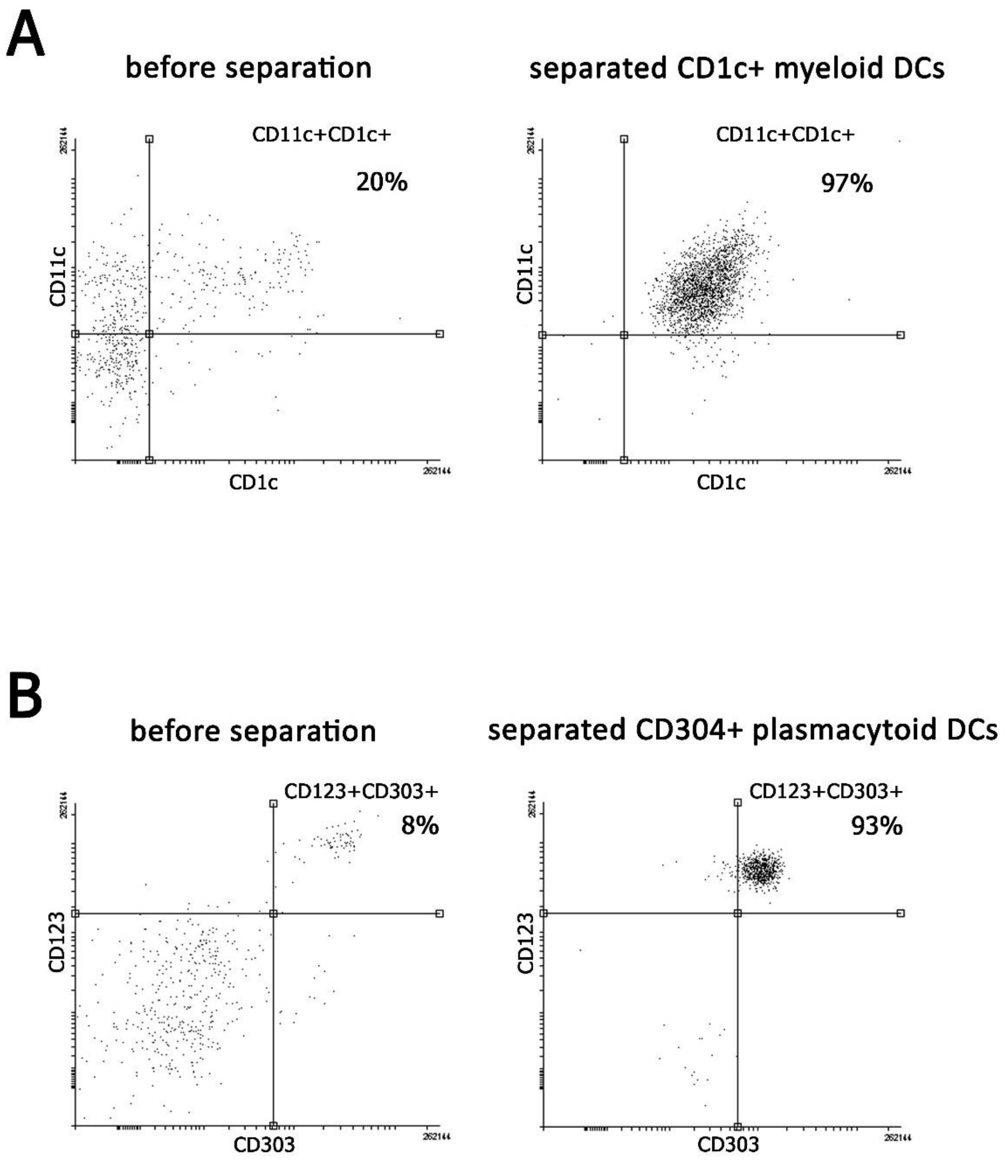

Supplementary Figure 3. Purity of magnetically separated DCs subpopulations. The analysis was performed with flow cytometry with the use of following gating on CD11c+ CD1c+ and CD123+CD303+ selectively identified mDCs (panel A) and pDCs (panel B), respectively.
